# The choice of region of interest after spinal procedures alters bone mineral density measurements

**DOI:** 10.1371/journal.pone.0285898

**Published:** 2023-05-16

**Authors:** Yen-Huai Lin, Che-Shi Chou, Michael Mu Huo Teng

**Affiliations:** 1 Department of Medical Imaging, Cheng Hsin General Hospital, Taipei, Taiwan; 2 Department of Medicine, School of Medicine, National Yang Ming Chiao Tung University, Taipei, Taiwan; Medical University of Vienna: Medizinische Universitat Wien, AUSTRIA

## Abstract

**Purpose:**

Vertebrae affected by artifacts, such as metallic implants or bone cement, should be excluded when measuring the spine bone mineral density (BMD) by dual-energy X-ray absorptiometry (DXA). Exclusion may be performed using two methods: first, the affected vertebrae are included in the region of interest (ROI) and subsequently excluded from the analysis; second, the affected vertebrae are completely excluded from the ROI. This study aimed to investigate the influence of metallic implants and bone cement on BMD with and without the inclusion of artifact-affected vertebrae in the ROI.

**Methods:**

DXA images of 285 patients, including 144 with spinal metallic implants and 141 who had undergone spinal vertebroplasty from 2018 to 2021, were retrospectively reviewed. Spine BMD measurements were performed when the images were evaluated using two different ROIs for each patient during the same examination. In the first measurement, the affected vertebrae were included in the ROI; however, the affected vertebrae were excluded from the BMD analysis. In the second measurement, the affected vertebrae were excluded from the ROI. Differences between the two measurements were evaluated using a paired t-test.

**Results:**

Among 285 patients (average age, 73 years; 218 women), spinal metallic implants led to an overestimation of bone mass in 40 of 144 patients, whereas bone cement resulted in an underestimation of bone mass in 30 of 141 patients when the first measurement was compared with the second measurement. The opposite effect occurred in 5 and 7 patients, respectively. Differences in results between the inclusion and exclusion of the affected vertebrae in the ROI were statistically significant (*p*<0.001). Spinal implants or cemented vertebrae included in the ROI might significantly alter BMD measurements. Additionally, different materials were associated with varying modifications in BMD.

**Conclusion:**

The inclusion of affected vertebrae in the ROI may notably alter BMD measurements, even when they are excluded from the analysis. This study suggests that the vertebrae affected by spinal metallic implants or bone cement should be excluded from the ROI.

## Introduction

Spinal procedures such as the use of metallic implants and vertebroplasty are common treatments for degenerative pathologies or osteoporotic fractures, and the rates of these procedures continue to increase worldwide [1,2]. In the United States, a continuous upward trend for spinal procedures has been observed, with an increase of 118% from 1998 to 2014 [3]. As the global aging population continues to increase, these procedures are expected to become more prevalent.

Dual-energy X-ray absorptiometry (DXA) is a standard tool used for assessing bone health. Owing to the DXA methodology, high-density materials such as metallic prostheses and bone cement may affect bone mineral density (BMD) measurements. In particular, the presence of high-density materials such as catheters, contrast agents, and spinal cord stimulators can increase BMD measurements [4–6]. Tantalum surgical clips, which appear black on DXA, can further decrease BMD measurements, whereas they appear white on radiography [4]. Artifacts in paraspinal soft tissues can also affect BMD measurements because of the differential attenuation by bone and soft tissue [4]. Thus, it is important to understand the influence of spinal procedures on BMD because artifacts can affect the precision and accuracy of these measurements.

The influence of metal-induced artifacts on DXA images has been investigated in several studies; for example, Giangregorio and Webber reported that whole body bone mass increased with an extraneous stainless steel spinal rod [7]. Hsiao et al. showed that spinal metallic artifacts led to a significant overestimation of bone mass of the trunk, whereas that of the extremities was not affected [8]. Therefore, spinal metallic implants could cause an overestimation of lumbar spine BMD. Most DXA system manufacturers mention that vertebrae affected by artifacts at L1–4 should still be included in the region of interest (ROI) and that the affected region should subsequently be excluded from the analysis of lumbar spine BMD and T-score. However, whether this method can eliminate the effects of spinal artifacts on BMD estimates remains unclear. Furthermore, the unaffected bone mass of the extremities indicates that metallic artifacts do not affect BMD estimates when the examined ROI does not include the region affected by artifacts [8]. Therefore, the influence of the ROI—including or excluding the vertebrae affected by artifacts—on lumbar spine BMD requires further investigation. Similar to metallic implants, bone cement for spinal vertebroplasty results in high-density artifacts; however, whether bone cement also leads to an overestimation of bone mass has not yet been explored in the literature.

According to the 2019 International Society for Clinical Densitometry (ISCD) Adult Official Positions, vertebrae affected by artifacts should be excluded [9]. Additionally, anatomically abnormal vertebrae may be excluded from the analysis if there is more than a 1.0 T-score difference between the vertebra in question and adjacent vertebrae. Exclusion may be performed using two methods: first, the affected vertebrae are included in the ROI and subsequently excluded from the analysis; second, the affected vertebrae are completely excluded from the ROI. The first method is the general procedure performed by densitometry technologists to comply with ISCD positions. In order to determine whether there is more than a 1.0 T-score difference between the vertebra in question and adjacent vertebrae and to exclude the questionable level from the analysis, technologists must include the vertebra in question in the ROI. Thus far, the first method has been performed in clinical practice, and related case reports has been published in the literature [6]. Therefore, the present study aimed to investigate the influence of spinal metallic implants and bone cement on lumbar spine BMD measurements, with the ROI including or excluding the vertebrae affected by artifacts. We hypothesized that the vertebrae affected by artifacts should be excluded from the ROI to eliminate the effects of artifacts on BMD.

## Materials and methods

### Patients

This retrospective hospital-based study was conducted from 2018 to 2021. DXA images of Chinese patients who underwent DXA and who received spinal implants or underwent vertebroplasty at any site from L1 to L4 were retrospectively reviewed. In accordance with the 2019 ISCD Adult Official Positions, patients who had only one evaluable vertebra due to different causes were excluded. A total of 285 patients were enrolled (S1 Fig). This study was approved by the Institutional Review Board (IRB) of Cheng Hsin General Hospital (IRB approval no.: (914)110A-60), and the requirement for the acquisition of informed consent from patients was waived owing to the retrospective nature of this study. All methods were performed in accordance with relevant guidelines and regulations.

### Spinal ROI

**Fig 1:**
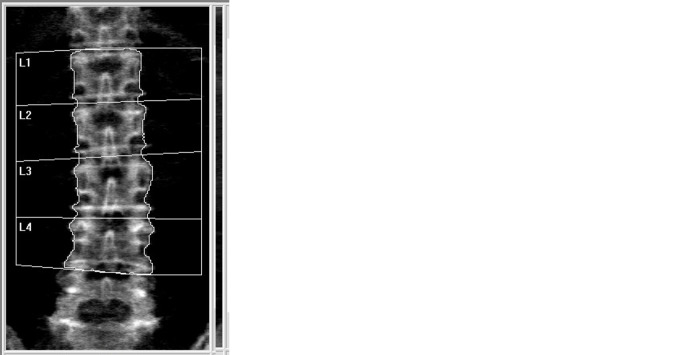
Representation of spine bone mineral density (BMD) data from a patient who underwent spinal vertebroplasty. **(a)** The inclusion of vertebrae affected by bone cement in the region of interest (ROI) for the computer analysis of BMD data, and the exclusion of affected vertebrae from the analysis of lumbar spine BMD; in this example, the affected vertebra was L2. **(b)** A situation in which the affected vertebrae were excluded from the ROI. **(c)** dual-energy X-ray absorptiometry image of the same patient before spinal vertebroplasty performed one year prior; this patient did not take any medicine that could affect bone health during the intervening time. The differences in BMD at L3–4 showed in Table 1.

The patients were divided into the spinal metallic implant and bone cement groups. Spine BMD measurements were performed by the same technician when the DXA images were evaluated using two different ROIs for each patient during the same examination. The first measurement included the affected vertebrae in the ROI but excluded them from the analysis of lumbar spine BMD and T-score, as shown in Fig 1A. When the affected vertebrae were included in the ROI, the BMD and T-score of the respective affected and non-affected vertebral bodies included in the ROI were calculated and displayed in the screen of the densitometer. The second measurement excluded the affected vertebrae from the ROI, as shown in Fig 1B. After the affected L2 vertebral body was excluded from the ROI, the BMD and T-score of the affected L2 vertebral body were not calculated and not displayed in the screen of the densitometer. Data on the area estimates, bone mineral content (BMC), and BMD were collected at both measurements in the same vertebrae.

From the above description, all vertebral levels included in the ROI would be calculated by the densitometer. After calculation, the BMC, BMD, and T-score of the respective vertebral levels and different combinations of vertebral levels included in the ROI would be displayed. The BMC, BMD, and T-score would not be calculated for levels excluded from the ROI. A densitometer technologist usually performed the process of L1-L4 vertebrae inclusion or exclusion from the ROI. Conversely, in the present study, the inclusion or exclusion from the BMD analysis meant reading of DXA data after proper lumbar spine levels were included in the ROI and deciding which combination of vertebral levels was to be selected to represent the patient’s spine T-score and BMD.

### BMD

Spine BMD was measured using a DXA scanner (Horizon W; Hologic Inc., Bedford, MA, USA). In accordance with the 2019 ISCD Adult Official Positions, 30 healthy patients were recruited to perform a precision analysis, and the least significant change (LSC) was calculated, with patient repositioning between scans carried out by the same technician [9]. The coefficient of variation was 0.94% for the lumbar spine BMD and 0.026 g/cm^2^for the LSC. The LSC was used to report the differences between the two measures. Precision errors with patient repositioning between scans had been shown to be greater than those without patient repositioning [8]. A stringent LSC with patient repositioning was used in this study. Greater changes in BMD measurements than the LSC were considered clinically significant.

### Statistical analysis

The two spine BMD measurements were used to compare the differences in area, BMC, and BMD in the presence and absence of the affected vertebrae in the spinal ROI. Difference between the two measurements in the same patient were evaluated using a paired t-test. The chi-square test was used to examine the association between changes in BMD and the two different materials used for spinal procedures—namely, spinal metallic implants and bone cement. All reported *p*-values were two-sided, and a *p*-value of <0.05 was considered statistically significant. Statistical analysis was performed using IBM SPSS Statistics for Windows version 19.0 (IBM Corp., Armonk, NY, USA).

## Results

Table 1 showed the differences in BMD of the representative patient who underwent spinal vertebroplasty in Fig 1. The difference between the BMD at L3–4 in Fig 1B (excluding the affected L2 vertebra from the ROI) and Fig 1C (DXA performed one year prior to vertebroplasty) was less than the LSC (0.026 g/cm^2^). However, the differences in BMD at L3–4 between Fig 1A (including the affected L2 vertebra in the ROI) and Fig 1B, and between Fig 1A and 1C were both greater than the LSC. The difference in BMD greater than LSC was most likely caused by the inclusion of the affected L2 vertebral body in the ROI in Fig 1A.

**Table 1:** Comparison of bone mineral densities in a representative patient shown in Fig 1.

	Fig 1A	Fig 1B	Fig 1C
BMD at L3–4 (g/cm^2^)	0.652 (D1)	0.725 (D2)	0.716 (D3)
Difference in BMD (D1-D2)	-0.073(> LSC*)		
Difference in BMD (D2-D3)		0.009 (< LSC*)	
Difference in BMD (D1-D3)			-0.064(> LSC*)

*The LSC in this study was 0.026 g/cm^2^.

A total of 285 patients (67 men and 218 women) were included in the present study. The mean age, height, weight, and body mass index were 73 years, 155.3 cm, 59.5 kg, and 24.6 kg/m^2^ respectively (Table 2). Among the included patients, 144 received spinal metallic implants, whereas 141 underwent vertebroplasty.

**Table 2:** Descriptive characteristics (n = 285).

Variable	Number of cases (*n*)	Percentage (%)	Mean	Standard deviation
Spinal procedures				
Metallic implant	144	50.5		
Vertebroplasty	141	49.5		
Gender				
Men	67	23.5		
Women	218	76.5		
Age (years)			73.3	9.5
Height (cm)			155.3	7.5
Weight (kg)			59.5	11.4
Body mass index (kg/m^2^)			24.6	4.3

Table 3 shows the differences between the two BMD measurements, in which the affected vertebrae were included or excluded in the ROI. In the metallic implant group, the BMC and BMD significantly increased, whereas the area was not affected. Thus, the inclusion of vertebrae affected by metallic implants in the ROI and their exclusion from the BMD analysis still resulted in an overestimation of bone mass. In contrast, in the bone cement group, the BMC and BMD significantly decreased, whereas the area was not affected. Therefore, including the cemented vertebrae in the ROI and excluding them from the BMD analysis led to an underestimation of bone mass.

**Table 3:** Differences between two bone mineral density measurements including or excluding the affected vertebrae in the region of interest.

	Metallic implant					Bone cement				
	Including affected vertebrae		Excluding affected vertebrae			Including affected vertebrae		Excluding affected vertebrae		
	Mean	Standard deviation	Mean	Standard deviation	*p*	Mean	Standard deviation	Mean	Standard deviation	*p*
Area (cm2)	29.8	6.5	29.8	6.5	0.561	34.5	8.0	34.6	8.1	0.081
Bone mineral content (g)	27.0	9.2	26.6	9.5	*p*<0.001	27.0	9.4	27.4	9.4	*p*<0.001
Bone mineral density (g/cm2)	0.894	0.18	0.880	0.17	*p*<0.001	0.771	0.16	0.782	0.16	*p*<0.001

Table 4 shows the association between the distinct materials used for spinal procedures and the varying modifications in BMD. The difference in BMD was greater than the LSC in 82 (29%) out of 285 patients. Among 144 patients with metallic implants, 40 (28%) showed an increased BMD greater than the LSC when the affected vertebrae were included in the ROI and excluded from the analysis, as compared with when they were excluded from the ROI. However, among 141 patients who underwent spinal vertebroplasty, 30 (21%) showed a decreased BMD greater than the LSC. Additionally, the inclusion of affected vertebrae altered the DXA diagnosis from osteoporosis to osteopenia in two patients, from osteopenia to osteoporosis in six patients, and from normal bone density to osteopenia in five patients. Furthermore, there was a significant difference between distinct materials, including metallic implants and bone cement for vertebroplasty, and the various modifications in BMD (*p* < 0.001). Both metallic implants and bone cement resulted in high-density artifacts on DXA; nevertheless, they had a diverse effect on BMD.

**Table 4:** Association between the distinct materials used for spinal procedures and various modifications in BMD when the affected vertebrae included in the ROI and excluded from the analysis are compared with the affected vertebrae excluded from the ROI.

	Metallic implant (n = 144)		Bone cement (n = 141)		
	No.	%	No.	%	*p* value for χ2
Bone mineral density					
No change	99	68.8	104	73.8	*p*<0.001
Significantly decreased	5	3.5	30	21.3	
Significantly increased	40	27.8	7	5.0	

## Discussion

Vertebrae affected by artifacts should be excluded according to the ISCD criteria. Most DXA system manufacturers state that the vertebrae affected by artifacts at L1–4 should be included in the ROI and that the affected region should subsequently be excluded from the analysis of lumbar spine BMD and T-score (Fig 1A). However, we found that the inclusion of such affected vertebrae in the ROI might still alter BMD measurements, suggesting that affected vertebrae should be excluded from the ROI (Fig 1B). In our study, the difference in BMD was greater than the LSC in 29% of patients. Furthermore, spinal metallic implants in 28% of patients led to an overestimation of bone mass, whereas bone cement in 21% of patients resulted in an underestimation of bone mass. Different materials were associated with varying modifications in BMD.

The presence of spinal metallic implants led to an overestimation of bone mass. Hsiao et al. assessed 30 patients with and without titanium spinal implants and found a significant increase in the trunk BMC (with the inclusion of metallic implants in the ROI) using a fan-beam DXA scanner [8], whereas estimates in the extremities were unaffected (without the inclusion of metallic implants in the ROI). Similarly, our study showed that the inclusion of vertebrae affected by spinal metallic implants in the ROI and their exclusion from the lumbar spine BMD analysis might still result in an overestimation of bone mass. The unaffected bone mass of the extremities supported our finding that the exclusion of the affected region from the ROI might have eliminated the effect of spinal metallic artifacts on BMD [8].

To the best of our knowledge, the influence of bone cement on BMD measurements has not yet been investigated in the literature. In this study, spinal BMC estimates were significantly lower when the affected vertebrae were included in the ROI among patients who underwent spinal vertebroplasty. Conversely, bone mass was underestimated, indicating that not all high-density artifacts led to an overestimation of bone mass. The bone cement for vertebroplasty comprised Plexiglas—polymethyl methacrylate was the most common. Polymethyl methacrylate is a strong, lightweight material with a density of 1.17–1.20 g/cm^3^ [10]. To make the cement radiopaque, a small amount of contrast agent—either zirconium dioxide or barium sulphate—is added [11]. In contrast to metallic implants, bone cement exerted a diverse effect on BMD, despite causing similar high-density artifacts; material differences may be the cause.

We propose that the solution to the problems caused by high-density artifacts is to exclude the affected regions. Half-body scans have been used to replace the unilaterally affected extremity with high-density artifacts with the contralateral normal extremity [12,13]. Although this method works well for artifacts in the extremities, it is unsuitable for those within the vertebrae. The iDXA system (GE®, Boston, MA, USA) has an automated software-based artifact detection (ASAD) component that removes image artifacts of non-tissue origin. Theoretically, removing implant artifacts from the analysis may reduce the influence of high-density artifacts. However, this method still includes affected vertebrae in the ROI and excludes them from the analysis, which will still alter BMD measurements, as we had demonstrated. Due to the differential attenuation by bone and soft tissue on DXA, BMD alterations in the bone affected by artifacts may alter the calculation of lumbar spine BMD and T-score [4]. Furthermore, Harper et al. reported that ASAD removed some, but not all, implant artifacts, resulting in an overestimation of BMD in older adults who had undergone total knee replacement [14]. Therefore, the exclusion of the affected vertebrae from the ROI may be a better solution to eliminate the influence of artifacts in patients undergoing spinal procedures.

This study has several strengths. First, to our knowledge, this is the first study to investigate the influence of endogenous, rather than extraneous, spinal artifacts on BMD measurements. Second, our results demonstrate the effect of bone cement on BMD measurements, which has not been previously explored. However, this study also has some limitations that should be noted. First, the influence of spinal artifacts may vary with the scanner used; thus, our results may only be applicable to Hologic scanners. Nonetheless, overestimation of BMD owing to artifacts resulting from total knee replacement has also been reported using iDXA [14]. Further studies are required to verify these results. Second, the effects of different amounts of spinal metallic implants or bone cement were not investigated. Third, the actual materials of which the metallic implants and bone cement were composed could not be determined. However, spinal implants are mostly made of titanium, and polymethyl methacrylate is the most widely used material for spinal vertebroplasty.

In conclusion, we showed that the inclusion of the affected vertebrae in the region of interest and their exclusion them from bone mineral density analysis may notably alter lumbar spine bone mineral density estimates, affecting 29% of patients. Our findings suggest that the region of interest should exclude affected vertebrae in patients with spinal metallic implants or bone cement.

## Supporting information

S1 Fig Flow diagram of enrolled patients. (DOCX)
