# Supplementary material for: The choice of region of interest after spinal procedures alters bone mineral density measurements
Source: PLoS One. 2023 May 16;18(5):e0285898. doi: 10.1371/journal.pone.0285898 (PMC10187904; doi:10.1371/journal.pone.0285898)
Supplement: S1 Fig — (DOCX) [file pone.0285898.s001.docx]

**S1 Figure.** Flow diagram of enrolled patients

**Retrospective reviewed patients who had undergone DXA and received spinal implants or vertebroplasty at any site from L1 to L4 from 2018 to 2021**

n = 287

**Excluded patients who had only one evaluable vertebra**

n = 2

**Enrolled patients**

n = 285

**Patients who received spinal metallic implants**

n = 144

**Patients who received spinal vertebroplasty**

n = 141
